# Prolonged sitting is not associated with altered shear‐mediated dilation of the internal carotid artery, despite impairing lower limb endothelial function

**DOI:** 10.14814/phy2.70097

**Published:** 2025-01-29

**Authors:** Shotaro Saito, Kento Dora, Marino Karaki, Narumi Kunimatsu, Hayato Tsukamoto, Jun Sugawara, Erika Iwamoto, Shigehiko Ogoh

**Affiliations:** ^1^ Department of Biomedical Engineering Toyo University Saitama Japan; ^2^ Graduate School of Health and Sport Sciences Chukyo University Aichi Japan; ^3^ Faculty of Sport Sciences Waseda University Saitama Japan; ^4^ Human Informatics and Interaction Research Institute National Institute of Advanced Industrial Science and Technology Ibaraki Japan; ^5^ School of Health Sciences Sapporo Medical University Sapporo Hokkaido Japan; ^6^ Neurovascular Research Laboratory University of South Wales Pontypridd UK

**Keywords:** cerebral blood flow, cerebrovascular shear rate, internal carotid artery, sedentarism

## Abstract

The present study aims to examine the effect of 4 h of continuous sitting on cerebral endothelial function, which is a crucial component of cerebral blood flow regulation. We hypothesized that 4 h of sitting may impair cerebral endothelial function similarly to how it affects lower limb vasculature. Thirteen young, healthy participants were instructed to remain seated for 4 h without moving their lower limbs. The blood flow and shear rate (SR) in the popliteal and internal carotid artery (ICA) were measured using duplex Doppler ultrasound. During the 4‐h sitting, peripheral (popliteal artery) and cerebral (ICA) endothelial function were assessed every hour. We induced peripheral and cerebral flow‐mediated dilation (pFMD and ICA FMD) using hyperemia (5 min of cuff inflation on lower limb, then deflation) or hypercapnia (30s of hypercapnia, end‐tidal partial pressure of CO_2_ + 9 mmHg), respectively. We then calculated each relative peak dilation from the baseline diameter to identify both pFMD and ICA FMD. We observed a significant decrease in pFMD starting at 2 h from the onset of sitting, and this reduction persisted throughout the 4‐h sitting [Base (6.8 ± 4.2%) vs. 2‐h (3.9 ± 2.0%), *p* = 0.044; vs. 3‐h (3.2 ± 1.8%), *p* = 0.016; vs. 4‐h (3.2 ± 1.9%), *p* = 0.005]. In contrast, during the 4‐h sitting, ICA blood flow, SR, and ICA FMD remained unchanged (*p* = 0.062, *p* = 0.068, and *p* = 0.203, respectively). Unlike peripheral endothelial function, cerebral endothelial function remained stable during 4‐h sitting. This suggests that the acute effect of prolonged sitting on cerebral vasculature differs from that of lower limb vasculature.

## 1 INTRODUCTION

Advancement in technology has influenced both work and leisure activities and has undoubtedly brought convenience to people's life and has significantly transformed lifestyles. However, technological progress has also led to extended sedentary periods potentially harming health. Importantly, a sedentary lifestyle (particularly chronic prolonged sitting) is associated with not only cardiovascular diseases (Biswas et al., 2015; English et al., 2016) and mortality risk (Chau et al., 2013) but also with cerebral disease (Arnardottir et al., 2016; Falck et al., 2017; Kyu et al., 2016). Habitual prolonged sitting has been linked to an increased risk of stroke (Kyu et al., 2016) and cerebral atrophy (Arnardottir et al., 2016). Therefore, an increased risk of diseases linked to a sedentary lifestyle, particularly cerebral diseases, which can contribute to the demand for nursing care, is a notable concern in contemporary society (Burton, 2000).

These findings suggest that chronic physical inactivity is a primary factor contributing to the elevated disease risk associated with habitual prolonged sitting (Kerr & Booth, 2022). However, recent epidemiological studies have revealed that even individuals who engage in regular exercise (i.e., more than 150 min of moderate‐intensity aerobic activity per week) face an elevated risk of diseases when their daily sitting time is prolonged (Kerr & Booth, 2022). This underscores the fact that prolonged sitting is an independent risk factor for various health issues (Kerr & Booth, 2022). It is well established that prolonged sitting leads to endothelial dysfunction, especially in the lower limbs, which serves as an indicator of peripheral vascular health (Credeur et al., 2019; Restaino et al., 2015; Thosaret al., 2015). Although there are some inconsistent findings, several previous studies have reported increased arterial stiffness in the central artery. This suggests that prolonged sitting can have a detrimental impact on central vascular health (Credeur et al., 2019). However, the exact physiological mechanism responsible for the increased risk of cerebral disease associated with prolonged sitting remains largely unknown.

Several previous studies have emphasized an association between prolonged sitting and the gradual reduction in cerebral blood flow (CBF) along with a decline in cognitive performance (Baker et al., 2018; Carter et al., 2018; Wheeler et al., 2019). While these studies primarily focused on elderly or middle‐aged participants, the findings suggest that prolonged sitting could potentially alter cerebral circulatory function independent of aging. Cerebral endothelial function is a fundamental aspect of cerebral circulation and has a vital role in maintaining brain homeostasis by regulating CBF through vasoconstriction and dilation, achieved via the production and release of vasoactive substances from endothelial cells (Green et al., 2017). Given the high metabolic rate of the brain and the presence of limited energy reserves, CBF regulation is crucial for ensuring an adequate supply of nutrients and oxygen. Importantly, animal studies have reported that dysregulation of CBF along with a decline in cerebral endothelial function in intracranial arteries is associated with cognitive dysfunction and increased risk of cerebral diseases such as dementia and stroke (Toda et al., 2009). Thus, cerebral endothelial function is considered a critical physiological factor in CBF regulation and for preserving brain function and health (Toda et al., 2009). Given these findings, it is imperative to investigate the impact of prolonged sitting on cerebral endothelial function in young, healthy participants to isolate its effects from those of aging and to precisely understand its influence on CBF regulation and cognitive function.

Also, assessing the cerebral endothelial functions over time during prolonged sitting may provide valuable insights into mitigating the risk of cerebral disease associated with prolonged sitting. A previous study demonstrated that prolonged sitting (3 h) decreased peripheral endothelial functions (Thosar et al., 2015); however, physiological changes in cerebral endothelial function are not similar to that of peripheral endothelial function (Suzuki et al., 2020). The study indicated that peripheral endothelial function is more sensitive to physiological alteration compared to cerebral endothelial function. The study also showed that cerebral homeostasis is likely stable for different physiological conditions; however, no study examined the effect of prolonged sitting on cerebral endothelial function. Thus, the aim of the present study was to identify the acute effect of continuous 4‐h sitting on cerebral endothelial function. Importantly, it is well known that aging decreases cerebral circulation (Lu et al., 2011) and function (Salthouse, 2009) and increases the risk of cardiovascular and cerebrovascular disease (North & Sinclair, 2012). Therefore, the effect of prolonged sitting on cerebral endothelial function could be modified by aging. To isolate the effect of prolonged sitting from the influence of aging, we decided to use young healthy participants in this study.

We hypothesized that if cerebral endothelial function decreased during prolonged sitting, then this suggested that habitual prolonged sitting could expose individuals to long‐term endothelial dysfunction, even in those individuals with normal endothelial function under control conditions. Therefore, this acute finding (although without any direct effects) may be clinically relevant, indicating that habitual daily prolonged sitting is associated with cardiovascular and cerebral diseases. Therefore, this result could be indicative of an acute response and offer insights into how prolonged sitting could contribute to long‐term cardiovascular and cerebral disease risk. For this purpose, we measured 30‐s hypercapnia stimulation‐induced flow‐mediated dilation (FMD) in the internal carotid artery (ICA), referred to as ICA FMD, every hour. A previous study (Hoiland et al., 2022) demonstrated that the infusion of the nitric oxide (NO) synthase inhibitor NG‐monomethyl‐l‐arginine reduced ICA FMD, suggesting that this novel transient hypercapnia test for cerebral shear‐mediated dilation in the ICA is largely endothelium‐derived NO‐dependent, despite the ICA being an extracranial artery. Moreover, the endothelium‐dependent vasodilatory response reflects the reactivity of its distal arteries (Pyke et al., 2008). Therefore, the endothelium‐dependent vasodilatory response of the ICA (i.e., ICA FMD) may provide insights into the cerebral vascular endothelial function of the anterior and middle cerebral arteries, which are distal arteries of the ICA and part of the anterior cerebral circulation. Assessing the ICA FMD response during prolonged sitting will help clarify the impact of prolonged sitting on cerebral endothelial function (Hoiland et al., 2022).

In the present study, we investigated the acute effect of prolonged sitting on endothelial function. If FMD is reduced during acute prolonged sitting, it suggests that habitual prolonged sitting repeatedly exposes to endothelial dysfunction for extended periods, even in those with normal endothelial function under controlled conditions. Importantly, acute changes in endothelial function can reflect baseline changes due to chronic effects (Dawson et al., 2018). Thus, these acute findings may have clinical relevance, as a sedentary lifestyle with habitual daily prolonged sitting is associated with an increased risk of cerebral and cardiovascular diseases. In lower limb circulation, it has been reported that prolonged sitting impairs endothelial function in the lower leg within just 1 h after sitting commences (Headid 3rd et al., 2020; Morishima et al., 2016; O'Brien et al., 2019; Park et al., 2022; Restaino et al., 2015, 2016; Vranish et al., 2017). Similarly, we hypothesized that a 4 h sitting may impair cerebral endothelial function and lead to cognitive impairment.

## 2 METHODS

### 2.1 Ethical approval

The protocol was approved by the Institutional Review Board of Toyo University (Approval Number: TU2022‐025) and complied with the standards outlined in the Declaration of Helsinki, except for registration in the database. All participants provided written informed consent prior to their participation.

### 2.2 Participants

This was a pilot study with five participants and subsequently confirmed sufficient statistical power (*n* = 8) using an effect size (f) of 0.75, significance level of 0.05, and power of 0.80 to detect a reduction in pFMD due to 4‐h sitting. Additionally, based on previous studies that indicate that prolonged sitting decreases CBF, we estimated the sample size using an effect size (η2) of 0.36, a significance level of 0.05, and a power of 0.80. Assuming a within‐participant correlation of 0.5, the calculated effective sample size (*n*) was 12 participants. Overall, 13 healthy participants (10 males and 3 females; age, 21 ± 1 years; height, 167 ± 8 cm; weight, 60 ± 11 kg; and body mass index, 22 ± 3 kg/m^2^) were recruited for this study. None of the participants had any known cerebrovascular or cardiovascular disease, were not taking medications, and were non‐smokers. Before the experiment, each participant was required to abstain from caffeine, alcohol, vitamin C supplementation, and strenuous exercise for 24 h. Moreover, given the potential effects of sleep deprivation and the stress of prolonged restraint, participants were instructed to maintain their normal sleep patterns before the study day to ensure an adequate sleep schedule and arrive at the laboratory on the day of the experiment.

Further, the participants were required to refrain from eating on the experimental day. All female participants (*n* = 3) underwent the experimental procedure during the early follicular phase (days 2–5) of their menstrual cycle. The experiments were started at 9:00 am, and the room temperature was maintained at 24–25°C. All participants had been awake for at least 2 h before the experiment.

### 2.3 Experimental procedure

The participants were required to visit the laboratory for 2 days within a week. During the first visit, all participants completed at least one practice session of the Go/No‐Go task (60 trials) and a memory recognition task (30 trials) to minimize learning effects.

On the day of the experiment (second visit), respiratory hemodynamics at baseline were initially assessed to evaluate ICA FMD. Cognitive performance (Go/No‐Go and memory recognition tasks) was assessed at baseline. Subsequently, baseline values were measured in the following order: (1) peripheral (popliteal artery) FMD (pFMD), (2) cerebrovascular hemodynamics and central arterial stiffness (carotid to femoral pulse wave velocity [cfPWV]) (measured simultaneously), and (3) ICA FMD. These baseline measurements were measured with all the participants being in the same resting position and condition as during the prolonged sitting period.

After completing these baseline measurements, participants were given a 15‐min break during which they consumed a light meal (i.e., one banana) and engaged in light stretching exercises (e.g., bending), considering the extended duration of the experiment. If necessary, participants used a restroom for urination and defecation. Following the break period, the participants were instructed to maintain a seated position for 4 h without moving their lower limbs. Based on previous studies indicating reduced cerebral blood flow due to prolonged sitting (Carter et al., 2018), participants were permitted to partake in cognitively undemanding desk‐based activities such as upper limb activities, watching television, or reading a book about 10 min before the measurement sessions.

**FIGURE 1:**
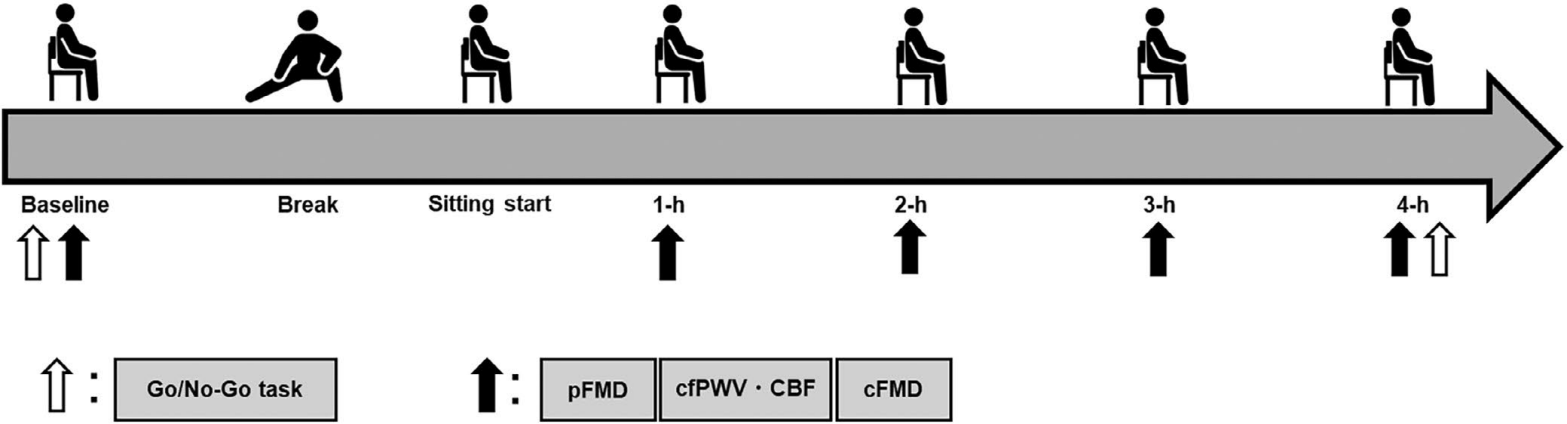
Experimental protocol in the present study.

Throughout the 4‐h sitting period, (1) pFMD, (2) cerebrovascular hemodynamics and cfPWV, and (3) ICA FMD were measured every hour. Following the end of the 4‐h sitting period, cognitive performance was assessed again. All measurements were conducted with the participants in a sitting position. All participants completed the full 4‐h sitting duration without any interruption (e.g., restrooms and dropouts) (Figure 1).

### 2.4 Cognitive performance

Executive function was evaluated using the Go/No‐Go task (Presentation ver. 21; Neuro Behavioral System, California, USA) (Akagi et al., 2019; Saito et al., 2021; Washio et al., 2021). In this task, a preparatory stimulus (green square) was displayed for 1 s on a computer screen in front of the participants. Following this, target stimuli (red and blue squares) or non‐target stimuli (yellow and purple squares) were displayed for 1 s. If the target stimuli appeared, the participants were instructed to press the left button of the computer mouse quickly using their right index finger. However, if non‐target stimuli were displayed, the participants were asked to withhold their responses. This task consisted of 60 trials with equal probabilities (30 target and 30 non‐target stimuli; total duration: 5 min). To assess executive function, the mean reaction time to target stimuli and the accuracy of the target or non‐target stimuli (Go and No‐Go accuracies, respectively) were calculated.

Memory recognition function was assessed using a memory recognition task (Presentation ver. 21) (Akagi et al., 2019; Saito et al., 2021; Washio et al., 2021). In this task, 30 Japanese words were presented on a computer screen at a rate of one word per second (total duration: 30 s), and the participants were instructed to memorize them. Subsequently, the participants completed the Go/No‐Go task (total duration: 5 min). As soon as the Go/No‐Go task was completed, 60 words were presented every 2 s (total duration: 2 min). During this phase, when the previously memorized 30 words (target stimuli) were displayed, participants were instructed to quickly press the left button of the computer mouse with their right index finger. However, if 30 non‐memorized words (non‐target stimuli) were presented, participants were asked to refrain from responding. To assess memory recognition function, the mean reaction time to target stimuli and the accuracy of 60 trials (words) were calculated.

### 2.5 Cardiorespiratory and cerebrovascular hemodynamics

All cardiorespiratory variables were measured hourly for 5 min. Heart rate (HR) was measured using a lead II electrocardiogram (PhysioFlow PF‐05 Lab1; Manatec Biomedical, Paris, France). Thoracic impedance (ΔZ), index of central blood volume, and stroke volume (SV) were assessed using impedance cardiography (PhysioFlow). Continuous beat‐to‐beat arterial blood pressure (ABP) was monitored using finger photoplethysmography (Finometer MIDI; Finapres Medical Systems, Amsterdam, Netherlands) with a cuff placed on the middle finger of the right hand at heart level. A Finometer cuff was attached only during the measurement sessions. To calibrate the beat‐to‐beat ABP for any fluctuations caused by reattaching the finometer cuff, systolic blood pressure (SBP) and diastolic blood pressure (DBP) values in the brachial artery were measured using automated blood pressure monitoring (Tango +; SunTech Medical, Oxfordshire, UK). Respiratory parameters, including minute ventilation (V_E_), tidal volume (V_t_), respiratory rate, and end‐tidal partial pressure of carbon dioxide (P_ET_CO_2_), were measured breath‐by‐breath using an automated gas analyzer (AE‐310S; Minato Medical Science, Osaka, Japan). Gas masks (Arco System, Chiba, Japan), required for assessing respiratory hemodynamics, were worn only during the measurement session. The data were continuously sampled at 1 kHz using an analog‐to‐digital converter (Power Lab 16 s; AD Instruments, Sydney, Australia) and stored on a laboratory computer for subsequent offline analyses.

Vascular diameter and time‐averaged mean blood velocity in the right ICA were randomly measured hourly for 30 s. A duplex Doppler ultrasound system (Vivid i; GE Medical Systems, Chicago, USA) equipped with a 13‐MHz linear array transducer was used for these measurements. The longitudinal image (diameter) and time‐averaged mean blood velocity were obtained using B (Brightness) and pulsed‐wave modes, respectively. The ICA was imaged 1.0–1.5 cm distal to the carotid bifurcation. The position of the linear array transducer was kept constant at an insonation angle of less than 60°. These variables were recorded in a video field at a frequency of 30 Hz using a video capture device (DVI2USB 3.0; Epiphan Systems, Ottawa, Canada) for subsequent offline analyses.

### 2.6 Central arterial stiffness

The cfPWV was determined by sequential acquisition of pressure waveforms from the right carotid and left femoral arteries using the same tonometer (SphygomoCor XCEL Pulse Wave Velocity [PWV]; AtCor Medical, Sydney, Australia). The pulse transit time from the carotid artery to the femoral artery (T_cf_) was obtained as the difference between the R wave on a simultaneously recorded ECG to waveform foot times. The distance between the carotid artery and suprasternal notch was measured on the body surface using a measuring tape and defined as the proximal distance (L_proximal_). The sum of the distances between the suprasternal notch and umbilicus and between the umbilicus and the common femoral artery was measured and defined as the distal distance (L_distal_) (Sugawara et al., 2021; Tomoto et al., 2023). Finally, the cfPWV was calculated using the following equation: cfPWV = (L_distal_ – L_proximal_)/T_cf_. Three evaluations were conducted during a single measurement session, and the average value was used as the representative measurement.

### 2.7 Peripheral endothelial function (pFMD test)

An inflating cuff was placed on the right lower leg. Vascular diameter and mean blood flow velocity in the right popliteal artery (PA) were initially measured at rest for 2 min to establish baseline values. Subsequently, a cuff positioned 10 cm distal to the knee joint was inflated at a pressure of 250 mmHg for 5 min to restrict blood flow in the calf. Following the release of cuff pressure, measurements were resumed and continued for 3 min. These variables were recorded in a video file at a frequency of 30 Hz using a video capture device (DVI2USB 3.0; Epiphan Systems, Ottawa, Canada) for subsequent offline analyses.

### 2.8 Cerebral endothelial function (ICA FMD test)

Cerebral endothelial function was assessed using transient hypercapnia intervention (lasting 30 s) to induce shear‐mediated ICA vasodilation response (referred to as ICA FMD), a method validated in previous studies (including our own) (Carr et al., 2020; Hoiland et al., 2017, 2022; Saito et al., 2023). The validity of this method for assessing cerebral endothelial function was established in a previous study that showed that the administration of a NO synthase inhibitor attenuates ICA shear‐mediated dilation (Hoiland et al., 2022). During the ICA FMD test, we measured the diameter and blood velocity of the right ICA, starting from a 2‐min resting baseline and continuing for 3 min after the onset of 30 s of transient hypercapnia stimulations. To induce hypercapnia, participants inhaled a mixed gas of room air and 100% CO_2_ from a mixing chamber (250 mL gas blender; Arco system, Chiba, Japan). The P_ET_CO_2_ was rapidly increased by approximately +9 mmHg from the resting baseline within approximately 5 s by manually adjusting the CO_2_ concentration in the mixing chamber. During the ICA FMD test, participants were guided to maintain a breathing pace of 20 breaths per minute using a metronome, and V_t_ was concurrently adjusted with visual feedback to keep V_E_ constant at the P_ET_CO_2_ value during spontaneous respiration. This instruction was employed to promptly and adequately manipulate the P_ET_CO_2_ values during the ICA FMD test because a higher respiratory rate could facilitate quicker breath‐by‐breath gas exchange (Carr et al., 2020; Hoiland et al., 2017, 2022). Some ICA FMD data were not measurable due to technical issues and were excluded (1‐h: *n* = 2, 2‐h: *n* = 1, 3‐h: *n* = 2).

### 2.9 Data analysis

#### 2.9.1 Cardiorespiratory and cerebrovascular hemodynamics

The ABP waveform, HR, V_E_, P_ET_CO_2_, ΔZ, and SV were analyzed offline using signal processing software (LabChart 8, ADInstruments). For ABP calibration, the average values of SBP and DBP over 30 s from the beat‐to‐beat ABP, required for the automated sphygmomanometer, were converted to match the unit of SBP and DBP values from the brachial blood pressure readings. Mean arterial pressure (MAP) was derived from the ABP waveform before calibration. Cardiac output (CO) was calculated by multiplying SV by HR. Total peripheral resistance (TPR) was calculated as MAP divided by CO.

The vascular diameter and time‐averaged mean blood velocity in the ICA were analyzed using custom‐designed edge‐detection and wall‐tracking software (version 2.0.4, S‐13037; Takei Scientific Instruments, Niigata, Japan). Shear rate (SR) and blood flow in the ICA were calculated from the measurement of each diameter and blood velocity [SR (/s) = 4 (blood viscosity constant) × blood velocity/diameter and blood flow (mL/min) = π × (diameter/2)^2^ × blood velocity × 60].

#### 2.9.2 pFMD test

The PA diameter and time‐averaged mean blood velocity were analyzed using custom‐designed edge‐detection and wall‐tracking software (version 2.0.4, S‐13037). PA data were detected using a previously reported algorithm (Black et al., 2008). The average values of the diameter and SR during the resting baseline were determined as the PA baseline diameter (PA D_base_) and mean SR (PA SR_base_). The peak values of the diameter and SR for 3 min after the release of the cuff pressure were identified as the PA peak diameter (PA D_peak_) and SR (PA SR_peak_). The pFMD was calculated using the following equation: pFMD (%) = (PA D_peak_ – PA D_base_)/PA D_base_ × 100. The SR area under the curve (SR_AUC_) was quantified as the area between the onset of hyperemia and the time of PA D_peak_. It was calculated using the trapezoidal rule as SR _AUC_ or blood flow _AUC_ (a.u.) = Σ [1/2 (*x*_*i* + 1_ − *x*_*i*_) (*y*_*i* + 1_ − *y*_*i*_) + (*x*_*i* + 1_ − *x*_*i*_) (*y*_*i*_ − *z*)], where *x* represents time, *y* represents PA SR or blood flow, and *z* represents PA SR or blood flow in the baseline. The pFMD normalized to SR (normalized pFMD) was calculated using the following equation: normalized pFMD (a.u.) = pFMD/SR_AUC_ × 1000.

#### 2.9.3 ICA FMD test

ICA diameter and time‐averaged mean blood velocity were analyzed using the same software as in the pFMD analysis (version 2.0.4, S‐13037). In cases where ICA diameter data were missing during the ICA FMD test owing to technical issues or swallowing, interpolation was performed using the least squares interpolation method. Subsequently, the ICA data were resampled at 1 Hz and underwent a two‐stage filtering process, which involved a median filter (with a rank of 7) followed by a Savitzky–Golay finite impulse response smoothing filter (with a window size of 13 data points and a polynomial order of 1) (Carter et al., 2016; Sakamoto et al., 2021). The median values of the ICA diameter and SR during the resting baseline were identified as the ICA baseline diameter (ICA D_base_) and SR (ICA SR_base_). The peak values of the diameter and SR for 3 min after transient hypercapnia stimulation were identified as the ICA peak diameter (ICA D_peak_) and SR (ICA SR_peak_). The ICA FMD was calculated using the following equation: ICA FMD (%) = (ICA D_peak_ – ICA D_base_)/ICA D_base_ × 100. The SR area under the curve (SR_AUC_) was quantified as the area between the onset of hypercapnia and time of the ICA D_peak_. It was calculated using the trapezoidal rule as SR _AUC_ or blood flow _AUC_ (a.u.) = Σ [1/2 (*x*_*i* + 1_ − *x*_*i*_) (*y*_*i* + 1_ − *y*_*i*_) + (*x*_*i* + 1_ − *x*_*i*_) (*y*_*i*_ − *z*)], where *x* represents time, *y* represents ICA SR or blood flow, and *z* represents ICA SR or blood flow in the baseline. The ICA FMD normalized to SR (normalized ICA FMD) was calculated using the following equation: normalized ICA FMD (a.u.) = ICA FMD/SR _AUC_ × 1000.

### 2.10 Statistical analysis

All data were presented as mean ± standard deviation (SD) or median (interquartile range; IQR) and analyzed using statistical software (SPSS Statistics ver. 27, International Business Machines, Tokyo, Japan). Normality of the data distribution was assessed using the Shapiro–Wilk test. The normally distributed data were analyzed using one‐way repeated‐measures ANOVA followed by a paired *t*‐test with Bonferroni correction, while non‐normally distributed data were analyzed using non‐parametric ANOVA (Friedman test) followed by Wilcoxon matched‐pairs tests with Bonferroni correction. Measurement variables with missing values were assessed using a linear mixed model. A *p‐*value <0.050 was considered as significant.

Accordingly, the effect sizes were calculated as eta squared (η2) for one‐way ANOVA outcomes, as Kendall's W for non‐parametric ANOVA outcomes, and as Wilcoxon matched‐pairs tests for r. Conservative interpretation guidelines for effect size were defined as small (η2 = 0.01, Kendall's W = 0.10 and *r* = 0.10), medium (η2 = 0.06, Kendall's W = 0.30 and *r* = 0.30), and large (η2 = 0.14, Kendall's W = 0.50 and *r* = 0.50) effects. The linear mixed model for effect size was not stated because it cannot be calculated.

## 3 RESULTS

### 3.1 Cardiovascular hemodynamics and function

**FIGURE 2:**
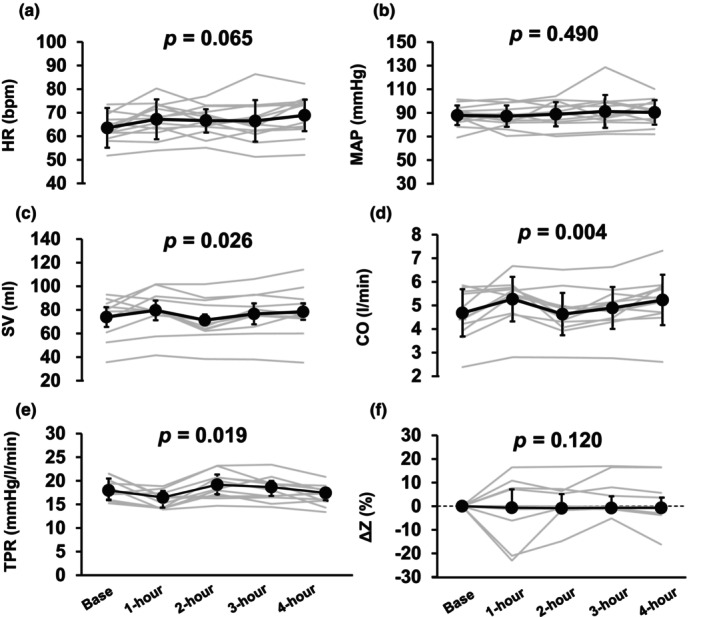
Heart rate (HR, a, all: *N* = 13), mean arterial pressure (MAP, b, all: *N* = 13), stroke volume (SV, c, base, 2‐h, 3‐h and 4‐h: *N* = 12 and 1‐h: *N* = 11), cardiac output (CO, d, base, 2‐h, 3‐h and 4‐h: *N* = 12 and 1‐h: *N* = 11), total peripheral resistance (TPR, e, base, 2‐h, 3‐h and 4‐h: *N* = 12 and 1‐h: *N* = 11), and thoracic impedance (ΔZ, f, base, 2‐h, 3‐h and 4‐h: *N* = 12 and 1‐h: *N* = 11) during 4‐h sitting. HR, MAP, SV, and CO values are mean ± standard deviation (SD). TPR and ΔZ values are median (interquartile range; IQR). HR and MAP were evaluated using one‐way repeated‐measures ANOVA. SV, CO, TPR, and ΔZ were evaluated using the liner‐mixed model.

During the 4‐h sitting period, HR (*p* = 0.065, η2 = 0.165), MAP (*p* = 0.490, η2 = 0.067), ΔZ (*p* = 0.120, Kendall's W = 0.141), and cfPWV (*p* = 0.736, η2 = 0.040, Figure 2 and Table 1) remain unchanged. In addition, although there was a significant time effect in SV, CO, and TPR (*p* = 0.026, *p* = 0.004, and *p* = 0.019, respectively), there was no difference in the baseline values during the 4‐h sitting (*p* > 0.449, *p* > 0.084, and *p* > 0.052, respectively, Figure 2).

**TABLE 1:** Carotid to femoral pulse wave velocity during 4‐h sitting period.

	Base	1‐h	2‐h	3‐h	4h	Main effect of time *p* value
cfPWV, m/s	5.9 ± 0.5	5.8 ± 0.9	5.8 ± 0.8	5.9 ± 0.8	6.0 ± 0.9	0.736

*Note*: cfPWV is expressed as mean ± standard deviation.

Abbreviation: cfPWV, carotid to femoral pulse wave velocity.

### 3.2 Cerebrovascular and respiratory responses

**FIGURE 3:**
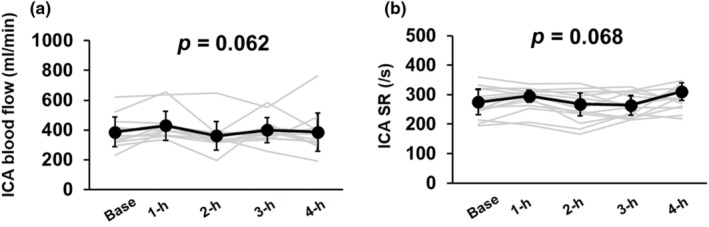
Blood flow and shear rate (SR) in the internal carotid artery (ICA, a, *n* = 13 and b, *n* = 13), during 4‐h sitting. All values are mean ± standard deviation (SD) or median (interquartile range; IQR). Blood flow in the ICA was evaluated using one‐way repeated‐measures ANOVA. SR in the ICA was evaluated using non‐parametric ANOVA.

During the 4‐h sitting periods, ICA blood flow (*p* = 0.062, η2 = 0.165), SR (*p* = 0.068, η2 = 0.162), and diameter (*p* = 0.062, Kendall's W = 0.133, Figure 3 and Table 2) remained unchanged. However, although the ICA velocity had a significant time effect (*p* = 0.004, Kendall's W = 0.299), it did not differ from the baseline during the 4‐h sitting period (*p* > 0.472, *r* < 0.055, Table 2). During the 4‐h sitting period, P_ET_CO_2_ did not change (*p* = 0.227, Table 2).

**TABLE 2:** Internal carotid, external carotid and vertebral artery and respiratory variable during 4‐h sitting period.

	Bas	1‐h	2‐h	3‐h	4‐h	Main effect of time *P* value
ICA diameter, mm	4.9 (4.7–5.1)	4.9 (4.8–5.2)	4.8 (4.5–5.1)	5.0 (4.7–5.2)	4.8 (4.5–5.0)	0.142
ICA velocity, cm/s	33.5 (30.1–33.5)	36.6 (37.0–34.3)	30.4 (29.9–34.8)	32.7 (31.9–36.2)	33.1 (31.5–37.6)	0.004
P_ET_CO_2_, mmHg	37.3 ± 3.3	38.2 ± 2.9	38.0 ± 2.8	38.2 ± 3.3	37.8 ± 2.5	0.227

*Note*: P_ET_CO_2_ is expressed as mean ± standard deviation. ICA diameter and velocity are expressed as median (interquartile range).

Abbreviations: ICA, internal carotid artery; P_ET_CO_2_, end‐tidal partial pressure of carbon dioxide.

### 3.3 Cognitive performance

The 4‐h sitting period did not change the reaction time, or Go accuracy or No‐Go accuracy of the Go/No‐Go task (*p* = 0.945, Cohen's d = 0.180, *p* = 1.000 and *p* = 1.000 *r* = 0.000, respectively, Table 3) or memory recognition task (*p* = 0.674, Cohen's d = 0.114 and *p* = 0.742, Cohen's d = 0.088, respectively).

**TABLE 3:** Cognitive performance before and after 4‐h sitting period.

	Pre	Post	*P* value
Go/No‐Go task			
Reaction time, ms	424 ± 53	424 ± 42	0.945
Go accuracy, %	100 ± 0	100 ± 0	1.000
No‐Go accuracy, %	100 (100–97)	100 (100–97)	1.000
Memory recognition task			
Reaction time, ms	833 ± 96	824 ± 98	0.674
Accuracy, %	79 ± 8	78 ± 9	0.742

*Note*: Reaction time in the Go/No‐Go task and reaction time and accuracy in the memory recognition task are expressed as mean ± standard deviation. Accuracy in the Go/No‐Go task is expressed as median (interquartile range).

### 3.4 pFMD

**FIGURE 4:**
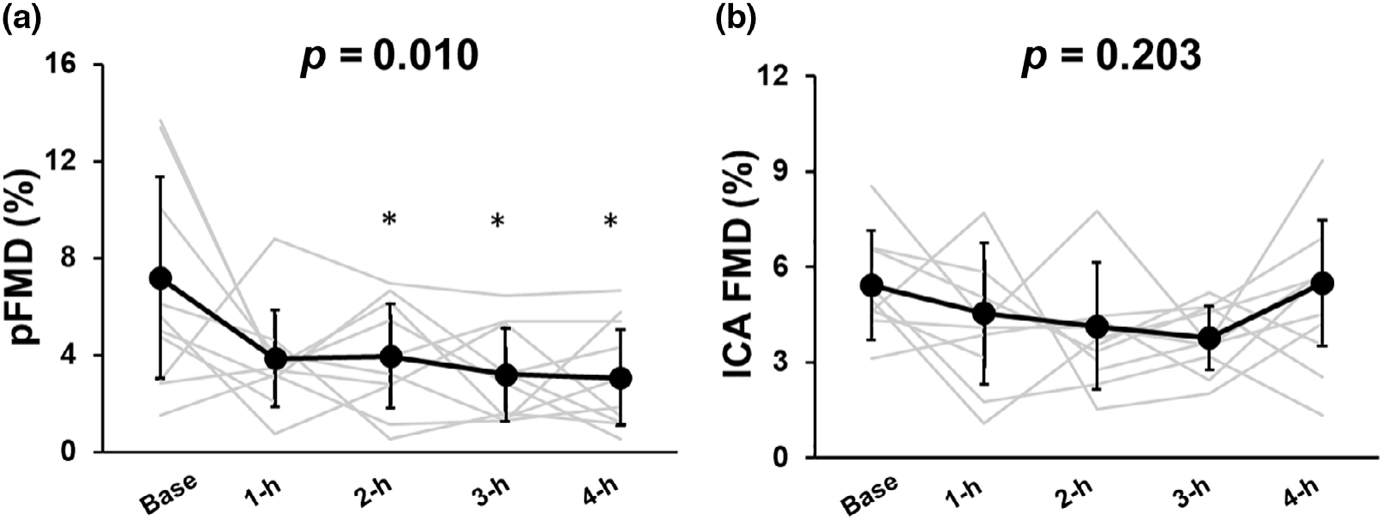
Flow‐mediated dilation in the popliteal artery (pFMD, a, baseline: *N* = 13, 1‐h: *N* = 11, 2‐h: *N* = 12, 3‐h: *N* = 11 and 4‐h: *N* = 13) and internal carotid artery (ICA FMD, b, baseline: *N* = 13, 1‐h: *N* = 11, 2‐h: *N* = 12, 3‐h: *N* = 12 and 4‐h: *N* = 13) during 4‐h sitting. All values are mean ± standard deviation (SD). pFMD and ICA FMD were evaluated using the liner‐mixed model. *p < 0.05.

The PA D_base_, D_peak_, SR_base_, SR_peak_, time to D_peak_, SR_AUC_, and blood flow_AUC_ did not change during the 4‐h sitting period (*p* = 0.134, *p* = 0.507, *p* = 0.717, *p* = 0.732, *p* = 0.655, *p* = 0.234, and *p* = 0.160, respectively; Table 4). There was a significant time effect on pFMD (*p* = 0.010, Figure 4a). It notably decreased at the 2‐h mark from the onset of sitting, and this reduction persisted throughout the 4‐h sitting period [base (6.8 ± 4.2%) vs. 2‐h (3.9 ± 2.0%), *n* = 8 decreased, *p* = 0.044; vs. 3‐h (3.2 ± 1.8%), *n* = 8 decreased, *p* = 0.016; vs. 4‐h (3.2 ± 1.9%), *n* = 10 decreased, *p* = 0.005]. Similarly, normalized pFMD also had a significant time effect (*p* = 0.005) and it decreased at the 1‐h mark from the onset of sitting, and this reduction persisted throughout the 4‐h sitting period [base (1.3 (0.5–1.6) a.u.) vs. 1‐h (0.5 (0.3–0.9) a.u.), *n* = 7 decreased, *p* = 0.009; vs. 2‐h (0.5 (0.3–0.9) a.u.), *n* = 8 decreased, *p* = 0.003; vs. 3‐h (0.3 (0.3–0.7) a.u.), *n* = 8 decreased, *p* = 0.001; vs. 4‐h (0.3 (0.2–0.5) a.u.), *n* = 10 decreased, *p* = 0.009].

**TABLE 4:** Results of the popliteal and internal carotid artery variables of flow‐mediated dilation test.

	pFMD						ICA FMD					
	Base	1‐h	2‐h	3‐h	4‐h	Main effect of time *P* value	Base	1‐h	2‐h	3‐h	4‐h	Main effect of time *P* value
D_base_, cm	4.9 ± 0.5	5.0 ± 0.5	4.9 ± 0.6	5.1 ± 0.6	5.1 ± 0.5	0.134	4.9 (4.7–5.2)	4.8 (4.6–5.3)	5.0 (4.8–5.2)	5.0 (4.7–5.2)	4.8 (4.6–5.0)	0.205
D_peak_, cm	5.2 ± 0.6	5.2 ± 0.6	5.1 ± 0.6	5.3 ± 0.6	5.3 ± 0.5	0.507	5.1 (4.7–5.4)	5.0 (4.8–5.5)	5.1 (5.0–5.4)	5.1 (4.9–5.3)	5.1 (4.9–5.3)	0.689
SR_base_, /s	36.8 ± 19.6	39.6 ± 18.1	37.4 ± 16.6	37.4 ± 15.6	34.0 ± 13.3	0.717	296.5 ± 82.9	268.3 ± 79.8	272.1 ± 55.9	259.5 ± 49.7	296.8 ± 62.8	0.556
SR_peak_, /s	162 (269–126)	173 (265–146)	227 (299–134)	175 (233–140)	189 (153–268)	0.732	439.3 ± 86.0	434.7 ± 53.2	364.1 ± 70.8	354.3 ± 64.6	394.5 ± 85.4	0.501
SR _AUC_, au	7819 ± 4758	11007 ± 9944	10091 ± 5558	9560 ± 2488	9087 ± 2726	0.655	2672 (1075–5651)	5358 (2869–6415)	2113 (973–6682)	2541 (1314–3827)	2650 (880–5171)	0.467
Blood flow _AUC_, au	11491 ± 4124	13536 ± 9508	12027 ± 5068	16758 ± 7946	12749 ± 2709	0.160	7819 ± 4758	7819 ± 4758	7819 ± 4758	7819 ± 4758	7819 ± 4758	0.337
Time to D_peak_, s	83 (50–99)	92 (88–101)	85 (46–106)	100 (71–127)	81 (51–92)	0.234	87 (79–124)	118 (108–137)	95 (81–138)	92 (73–111)	97 (70–105)	0.390
Normalized FMD, au	1.3 (0.5–1.6)	0.5 (0.3–0.9)*	0.3 (0.3–0.7)*	0.2 (0.2–0.6)*	0.3 (0.2–0.5)*	0.005	1.7 (0.9–5.0)	1.3 (0.7–1.5)	1.8 (0.5–2.7)	1.6 (0.9–2.2)	1.5 (1.0–3.3)	0.701

*Note*: D_base_, D_peak_, SR_base_, SR_AUC_ and blood flow_AUC_ of pFMD and SR_base_, SR_peak_ and blood flow_AUC_ of ICA FMD are expressed as mean ± standard deviation. SR_peak_, time to D_peak_ and normalized FMD of pFMD and D_base_, D_peak_, SR_AUC_, time to D_peak_ and normalized FMD of ICA FMD are expressed as median (interquartile range). pFMD, flow‐mediated dilation in the popliteal artery; ICA FMD, flow‐mediated dilation in the internal carotid artery; D_base_, baseline diameter in the popliteal artery or internal carotid artery; D_peak_, Peak diameter in the popliteal artery or internal carotid artery; SR_base_, baseline mean shear rate in the popliteal artery or internal carotid artery external carotid artery; SR_peak_, peak mean shear rate in the popliteal artery or internal carotid artery; SR_AUC_, SR area under the curve from onset hyperemia or hypercapnia to peak dilation; blood flow_AUC_, blood flow area under the curve from onset hyperemia or hypercapnia to peak dilation; normalized FMD, FMD normalized to the SR (FMD/SR_AUC_ × 1000).

* *p* < 0.050 versus base.

### 3.5 ICA FMD

The ICA D_base_, D_peak_, SR_base_, SR_peak_, time to D_peak_, SR_AUC_, and blood flow_AUC_ did not change during the 4‐h sitting period (*p* = 0.205, *p* = 0.689, *p* = 0.556, *p* = 0.501, *p* = 0.467, *p* = 0.390, and *p* = 0.337, respectively, Table 4). During the 4‐h sitting period, ICA FMD remained consistent (*p* = 0.203, Figure 4b). When adjusting ICA FMD using SR_AUC_, the normalized ICA FMD also remained consistent during the 4‐h sitting period (*p* = 0.701, Table 4).

## 4 DISCUSSION

To our knowledge, this is the first study to investigate the impact of prolonged sitting on cerebral endothelial function. Similar to previous studies (Restaino et al., 2015; Thosar, Bielko, Mather, et al., 2015), a 4‐h sitting period impaired peripheral (lower limb) endothelial function, whereas the cerebral endothelial function remained unchanged. This finding suggested that the acute effect of prolonged sitting on cerebral endothelial function differed from that of peripheral endothelial function in young, healthy individuals.

### 4.1 Effect of prolonged sitting on peripheral and cerebral endothelial function

Similar to previous studies (Restaino et al., 2015; Thosar et al., 2015), the present study found that 4 h of sitting attenuated peripheral (lower limb) endothelial function, a component of the cardiovascular regulatory system, in healthy, young participants (Figure 4a). Low and oscillatory shear stress can impair vascular endothelial function because this type of shear stress generates oxidative stress, a recognized risk factor for endothelial dysfunction (Tremblay et al., 2019). A seated position reduces blood flow and shear stress in the lower limbs compared to the supine or recumbent positions (Morishima et al., 2016; Restaino et al., 2016), and a decrease in shear rate in the peripheral artery has been observed throughout prolonged sitting (Thosar, et al., 2015). Interestingly, previous studies have reported that strategies to increase blood flow and shear stress in the lower limbs, such as heating and fidgeting, and administering antioxidants such as vitamin C, restored acute prolonged sitting‐induced decreases in pFMD (Morishima et al., 2016; Restaino et al., 2016; Thosar et al., 2015) to baseline levels. Therefore, the one physiological mechanism of acute prolonged sitting‐induced lower limb endothelial dysfunction is thought to be linked to a decrease in shear rate (Pekas et al., 2023).

Consistently, our findings showed that cerebral endothelial function remained unaffected during acute prolonged sitting, representing a different response than the peripheral endothelial function (Figure 4b). In the present study, the ICA SR was maintained during the 4‐h sitting period. Since decreasing SR is a risk factor for endothelial dysfunction, preservation of the ICA SR may have contributed to maintaining ICA FMD during the 4‐h sitting period. Thus, the difference in FMD between cerebral and peripheral arteries during prolonged sitting may be attributed to differences in shear rate response. Another significant mechanism that could explain the differing acute FMD responses to prolonged sitting between cerebral and peripheral arteries is blood viscosity, which increases after 2 h of sitting (Hitosugi et al., 2000). Additionally, increased muscle sympathetic nerve activity, which is higher during upright sitting compared to supine positions (Ng et al., 1995; Padilla et al., 2010), may also contribute. Also, prolonged sitting limits muscle pump influence and attenuated venous return, which affects blood flow regulation (Horiuchi & Stoner, 2021). However, in the present study, central blood volume (ΔZ) was unchanged during 4‐h sitting (Figure 2f), indicating that venous return was not limited by prolonged sitting. Yet, it remains unclear whether or how blood viscosity, increased sympathetic nerve activity, and reduced venous return during prolonged sitting affect cerebral arteries differently compared to peripheral arteries.

Importantly, this observation of different FMD responses to prolonged sitting between cerebral and peripheral arteries suggested that cerebral endothelial function may be more robust compared to that of the peripheral (lower limb) vasculature. Similarly, our previous study that investigated cerebral and peripheral endothelial function in smokers reported no change in cerebral endothelial function despite a decrease in pFMD (Suzuki et al., 2020). The robust cerebral endothelial function may be crucial for human homeostasis, given the brain's status as a vital organ.

### 4.2 Cerebral and peripheral endothelial function in relation to prolonged sitting‐induced disease risk

Importantly, the findings of the present study only demonstrate the acute effects of prolonged sitting. Therefore, it remains unclear whether this acute FMD response to prolonged sitting is associated with a habitual prolonged sitting‐induced increase in the risk of both cerebral and cardiovascular diseases. In this study, prolonged sitting led to an acute decrease in peripheral endothelial function (pFMD, Figure 4a). Endothelial dysfunction, which is crucial for atherosclerosis development (Green et al., 2011; Widlansky et al., 2003), is linked to a high risk of cerebral and cardiovascular diseases (Dormandy et al., 1999; Lorenz et al., 2007). However, acute prolonged sitting did not induce arterial stiffness in the central artery (cfPWV, Table 1). Evidence from meta‐analyses (Wilmot et al., 2012) and epidemiological studies (Warren et al., 2010) has shown that habitual prolonged sitting can increase the risk of cardiovascular disease. Consequently, prolonged habitual sitting may chronically expose individuals to daily endothelial dysfunction over a long period, potentially increasing the risk of disease.

Contrary to expectations, cerebral endothelial function remained unchanged during a 4‐h sitting period despite decreased peripheral endothelial function (Figure 4b), suggesting that any increased risk of cerebral disease that was associated with prolonged sitting (Arnardottir et al., 2016; Falck et al., 2017; Kyu et al., 2016) may not be due to impaired cerebral blood flow regulation. However, it is unclear whether habitual prolonged sitting reduces cerebral endothelial function. Arterial stiffness in the central artery affects cardiovascular health (Cecelja & Chowienczyk, 2009) and may lead to cerebral arteriosclerosis and disease (Hirata et al., 2006; van Sloten et al., 2015). Brain health is dependent on systemic circulatory health and not only cerebral function (van Sloten et al., 2015). While decreased peripheral arterial stiffness may relate to cerebral disease risk, acute prolonged sitting did not alter central arterial stiffness. Therefore, the mechanism behind the increased risk of cerebral disease induced by habitual prolonged sitting remains unclear. Further investigation is needed to understand how acute prolonged sitting affects long‐term vascular disease risk and its impact on cerebral circulation, lower limbs, and cerebral endothelial function.

Additionally, ICA FMD is known to be influenced by NO‐independent physiological factors, such as blood pressure (Hoiland et al., 2022). Moreover, MAP did not increase during the 30‐s CO_2_ inhalation test to assess ICA FMD (baseline; 90 ± 3 mmHg vs. during 30‐s CO_2_ inhalation; 90 ± 3, *p* = 0.921). On the other hand, shear stress induces constriction of intracranial arteries (not the ICA) under normal physiological pressures (Koller & Toth, 2012; Madden & Christman, 1999) and may suppress the shear‐mediated dilation of the intracranial arteries. In this regard, although ICA shear‐mediated dilation is expected to reflect the shear‐mediated dilation of intracranial vessels, further detailed investigation is required to understand the differences in the mechanisms contributing to the dilation.

### 4.3 Effect of prolonged sitting on CBF and cognitive performance

Since CBF regulatory function can influence cerebral circulatory homeostasis and cognition, we assessed CBF and cognitive function during prolonged sitting. Previous studies have shown that cerebral endothelial dysfunction is linked to reduced resting CBF (White et al., 1998) and cognitive impairment (de la Torre & Aliev, 2005; Toda et al., 2009). We hypothesized that prolonged sitting would acutely impair cerebral endothelial function and that CBF and cognitive function might be altered during prolonged sitting. However, contrary to our hypothesis, prolonged sitting did not decrease ICA blood flow or cognitive function in young, healthy participants (Figure 3 and Table 3).

Interestingly, previous studies have reported inconsistent findings, noting decreased CBF in sitting positions due to prolonged sitting (Carter et al., 2018; Maasakkers et al., 2020; Perdomo et al., 2019; Wheeler et al., 2019). Similarly, studies on the effect of prolonged sitting on cognitive function have yielded inconclusive results; cognitive function was either attenuated (Chrismas et al., 2019; Wheeler et al., 2020) or unchanged (Bergouignan et al., 2016; Wennberg et al., 2016) following prolonged sitting. These studies predominantly involved older and middle‐aged individuals, suggesting that aging may have influenced the outcomes. In contrast, our study specifically investigated the effects of prolonged sitting on cerebral homeostasis in young, healthy individuals to minimize the influence of aging factors. Further, we did not observe changes in ICA FMD, CBF, or cognitive function due to prolonged sitting. Therefore, aging might be an important risk factor, especially attenuation in cerebral homeostasis, (regulatory mechanism and cognition, etc.), for increased susceptibility to cerebral disease.

## 5 LIMITATIONS

The present study has some potential limitations. First, the female participants were exclusively in the early follicular phase and small sample size, consisting of only three individuals (not sex‐matched), compared with the male participants. Consequently, it remains unclear whether sex differences or the menstrual cycle affect FMD responses during the 4‐h sitting period. Second, the present study exclusively includes healthy young participants. As a consequence, the findings may not be generalizable to older individuals and females except for females in the early follicular phase and others with different characteristics. Third, the Go/No‐Go task used to evaluate the executive function was straightforward. Therefore, there is a concern that alterations in cognitive function due to prolonged sitting may not have been sufficiently detected. Fourth, we assessed cerebral circulatory function in the anterior circulation in response to prolonged sitting. Therefore, the present findings related to cerebral endothelial function within the anterior circulation in response to prolonged sitting might not be able to be extrapolated to the entire cerebral circulation (e.g., posterior circulation). Fifth, to ensure clarity and accuracy in evaluating the temporal response of each artery type, we opted to assess the change in FMD separately for each artery during prolonged sitting. Therefore, integrating their data and performing a joint analysis was not performed. Finally, in the present experimental protocol, we cannot rule out the possibility that the FMD response during prolonged sitting may have been influenced by variable factors such as the FMD stimulus, diurnal changes, measurement error, or natural variability from repeated measures. To address this, we conducted an additional control experiment with six new participants (4 males and 2 females; age 22 ± 1 years; height 172 ± 6 cm; weight 59 ± 7 kg; and body mass index 20 ± 1 kg/m^2^) who underwent 4 h of free activity with 2‐min walking breaks every 30 min, without prolonged sitting. In addition, we analyzed both groups (prolonged sitting and control condition) combined using a linear mixed model two‐way ANOVA. As a result, significant interaction effects were found for pFMD and normalized pFMD (*p* = 0.042 and *p* = 0.004). Specifically, pFMD and normalized pFMD decreased at the 3‐ and 1‐h mark from the onset of sitting, and this reduction persisted throughout the 4‐h sitting period (pFMD: base vs. 3‐h; *p* = 0.012 and vs. 4‐h; *p* = 0.005, normalized pFMD: base vs. 1‐h; *p* = 0.017, vs. 2‐h; *p* = 0.005, vs. 3‐h; *p* = 0.003 and vs. 4‐h; *p* = 0.001). In contrast, these measures remained stable over the 4 h of free activity (*p* > 0.962 and *p* > 0.200, respectively). On the other hand, ICA FMD and normalized ICA FMD remained stable throughout both the 4‐h sitting period and the free activity period (main effect of time; *p* = 0.779 and *p* = 0.904, respectively). Importantly, previous research has shown a decrease in pFMD after only 1 h of prolonged sitting, with no such decrease observed in the control conditions (prolonged sitting with walking breaks) (Thosar et al., 2015). This consistency with the present results, including the control condition, suggests that (1) pFMD observed during the prolonged sitting period is likely due to the 4‐h sitting itself, and (2) potential effects such as acclimatization to the FMD stimulus, diurnal changes, measurement error, or natural variability from repeated measures are likely to be minimal. However, it is important to fully acknowledge that the control condition experiments had a small sample size (*n* = 6) and involved different participants compared to the present study (4 h sitting).

## 6 CONCLUSION

Prolonged sitting leads to a decline in peripheral endothelial function, whereas cerebral endothelial function remains unaffected in young, healthy adults. This suggests that the immediate impact of prolonged sitting on the cerebral vasculature differs from that on the peripheral vasculature in young, healthy adults.

## AUTHOR CONTRIBUTIONS

S.S. and S.O. conceptualized and designed the research; S.S., K.D., M.K., N.K., and H.T. performed experiments; S.S. analyzed data; S.S. J.S., E.I., and S.O. interpreted the results of experiments; S.O. prepared figures; S.S. and S.O. drafted the manuscript; all authors edited and revised the manuscript. All authors approved the final version of the manuscript.

## FUNDING INFORMATION

S.S. is supported by a JST SPRING [grant number JPMJSP2159]. S.O. is supported by Grant‐in‐Aid for Scientific Research [grant number 22H003470] from the Japanese Ministry of Education, Culture, Sports, Science, and Technology.

## CONFLICT OF INTEREST STATEMENT

The authors declare that they have no conflict of interest.

## ETHICAL APPROVAL

All testing procedures were approved by the Institutional Review Board at Toyo University (Approval Number: TU2022‐025).

## CONSENT TO PARTICIPATE

Participants provided written informed consent before participation under the principles of the Declaration of Helsinki.

## CONSENT TO PUBLISH

Participants cannot be individually identified from the data published in this manuscript. Participants were made aware of the intent to publish this data when providing informed consent.

## Data Availability

The datasets generated and analyzed in the present study are available from the corresponding author upon reasonable request.
